# A Quantitative Point-of-Need Assay for the Assessment of Vitamin D_3_ Deficiency

**DOI:** 10.1038/s41598-017-13044-5

**Published:** 2017-10-26

**Authors:** S. Vemulapati, E. Rey, D. O’Dell, S. Mehta, D. Erickson

**Affiliations:** 1 Sibley School of Mechanical and Aerospace Engineering, Cornell University, Ithaca, NY 14853 USA; 2 School of Applied and Engineering Physics, Cornell University, 526 Campus Road, 409 Weill Hall, Ithaca, NY 14853 USA; 3 Institute for Nutritional Sciences, Global Health, and Technology (INSiGHT), Cornell University, Ithaca, NY 14853 USA; 4 Division of Nutritional Sciences, Cornell University, Ithaca, NY 14853 USA

## Abstract

Vitamin D is necessary for the healthy growth and development of bone and muscle. Vitamin D deficiency, which is present in 42% of the US population, is often undiagnosed as symptoms may not manifest for several years and long-term deficiency has been linked to osteoporosis, diabetes and cancer. Currently the majority of vitamin D testing is performed in large-scale commercial laboratories which have high operational costs and long times-to-result. Development of a low-cost point-of-need assay could be transformative to deficiency analysis in limited-resource settings. The best biomarker of vitamin D status, 25hydroxyvitamin D_3_ (25(OH)D_3_), however, is particularly challenging to measure in such a format due to complexities involved in sample preparation, including the need to separate the marker from its binding protein. Here we present a rapid diagnostic test for the accurate, quantitative assessment of 25(OH)D_3_ in finger-stick blood. The assay is accompanied by a smartphone-assisted portable imaging device that can autonomously perform the necessary image processing. To achieve accurate quantification of 25(OH)D_3_, we also demonstrate a novel elution buffer that separates 25(OH)D_3_ from its binding protein in situ, eliminating the need for sample preparation. In human trials, the accuracy of our platform is 90.5%.

## Introduction

Vitamin D refers to a group of fat soluble secosteroids responsible for promoting absorption of calcium, phosphate and zinc in the body^1,2^. It is also essential for the healthy growth and maintenance of bone and muscle^[Bibr CR3][Bibr CR3]^. Vitamin D deficiency causes rickets in children^[Bibr CR4][Bibr CR4]^ and low levels of vitamin D have been attributed to diabetes, heart disease, cancer and osteoporosis in adults^[Bibr CR5][Bibr CR5]^. Vitamin D is also critical for ensuring optimal immune function; for example, vitamin D is required for producing antimicrobial peptides such as cathelicidin, necessary to combat pathogens such as *Mycobacterium tuberculosis* in human macrophages^[Bibr CR6][Bibr CR6]^. Deficiency is defined by evaluating the serum concentrations of 25-hydroxyvitamin D, or 25(OH)D, a pro-hormone that is produced in the liver by hydroxylation of cholecalciferol, the biologically inert form of vitamin D that is produced in the skin. 25(OH)D is the major circulating form of vitamin D and is considered the best indicator of holistic vitamin D status. Supplemental vitamin D is available in two forms: ergocalciferol (vitamin D_2_) and cholecalciferol (vitamin D_3_). Even though commonly prescribed supplements contain either vitamin D_2_ or vitamin D_3_, it has been demonstrated that cholecalciferol has a higher efficacy at raising the total serum 25(OH)D status in individuals^7,8^. As a result, many of the supplements now on the market contain cholecalciferol. The Endocrine Society guidelines suggest that individuals with serum concentration of 25(OH)D greater than 75 nmol/L can be considered healthy while those with values between 50 and 75 nmol/L are insufficient and lower than 50 nmol/L are deficient^[Bibr CR9][Bibr CR9]^.

In the United States, the prevalence of vitamin D deficiency rate is estimated at 42%, with a higher incidence amongst the African American (82%) and Hispanic (69%) population^[Bibr CR10][Bibr CR10]^. Deficiency can be corrected with supplementation and dietary changes particularly when detected early; however, many people are unaware of their status due to the lack of easy to access methods for quantifying 25(OH)D_3_ levels in human serum. Point-of-care vitamin D deficiency testing remains a significant challenge due to the presence of vitamin D binding protein, a carrier protein that helps transport 25(OH)D in blood. 95–99% of available 25(OH)D in blood is bound to vitamin D binding protein^11,12^ and due to the nature of the strong hydrophobic bond between them, it is not possible to assess an individual’s vitamin D status without separating the two from each other. Furthermore, vitamin D binding protein is always present in significant molar excess in blood and has no correlation with the amount of circulating 25(OH)D^13–15^. As a result, it cannot be used as a target itself, a scheme which has been adopted for targets such as vitamin A^16–19^.

Current clinical methods for quantitation of 25(OH)D can be broadly divided into chromatographic methods with (LC/MS-MS: Liquid Chromatography Tandem Mass Spectrometry) and without (HPLC: High Performance Liquid Chromatography) mass spectrometric detection and competitive immunoassays such as RadioImmunoAssay (RIA). These techniques are expensive and time consuming due to the need for specialized equipment and personnel and the necessity to separate and remove vitamin D binding protein presents further complications by requiring elaborate sample preparation. Usually the sample is treated and incubated (for ~1 hour) with large amounts of organic solvents such as acetonitrile to perform the separation step. Further, organic solvents cannot be used directly with blood as they cause lysis of blood cells, and require that the blood be centrifuged into serum. Standard protocols also require an additional centrifugation step after the organic solvents are added in order to remove the precipitated protein. These tedious sample preparation requirements combined with the need for specialized equipment pose a severe limitation for the development of point-of-need tests for the assessment of vitamin D status.

In recent years, several research groups have employed microfluidic techniques to develop low cost methods to assess micronutrient status at the point-of-need. A microfluidic chip that was able to perform the detection of ferritin and CRP in human serum samples was demonstrated by Kartalov *et al*.^[Bibr CR20][Bibr CR20]^. Similarly, Dimov *et al*. exhibited the “stand-alone self-powered integrated microfluidic blood analysis system (SIMBAS)” for detection of vitamin B_6_^[Bibr CR21][Bibr CR21]^. While these works have demonstrated excellent proof of concepts for use at the point of care, they are limited by their inability to perform blood processing on chip and the need for expensive imaging equipment. Lee *et al*.^[Bibr CR22][Bibr CR22]^ demonstrated the vitamin AuNP-based Immunoassay Device (vitaAID), a system for quantification of vitamin D levels using a smartphone. In that work, a novel gold nanoparticle based immunoassay architecture was developed and analyzed by a smartphone camera, enabling quantification of serum samples without the need for expensive lab equipment. Even though the method helped transition vitamin D testing out of the lab, the technique was limited by its inability to perform blood processing on chip and non-trivial incubation time (~6 hours).

In this work, we present a rapid diagnostic test that is capable of accurate, quantitative assessment of vitamin D status. Our test focuses on quantification of 25(OH)D_3_, the largest component of circulating 25(OH)D, in the deficient range ( < 50nmol/L), as that is where the greatest need is. Quantitative ability is necessary in the deficient range to help distinguish between individuals who are severely and moderately deficient. In order to maintain compatibility with existing manufacturing techniques, we developed our diagnostic test as a lateral flow assay. Furthermore, our diagnostic test is integrated into a smartphone-assisted imaging platform which allows for easy operation and hassle-free access to test results. In addition to being capable of processing blood on strip, our test incorporates a novel elution buffer that is compatible with lateral flow assays to achieve separation of 25(OH)D_3_ from its binding protein *in situ*, eliminating the need for any sample preparation. We show reliable quantification of physiologically relevant vitamin D_3_ levels in standard solutions, commercial calibrators, human serum and finger stick blood.

## Results

**Figure 1:**
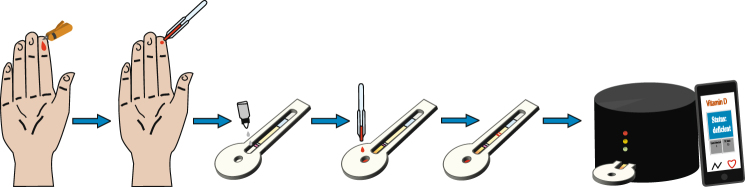
Protocol for Vitamin D_3_ diagnostic test. The TIDBIT is depicted as the last image on the right.

**Figure 2:**
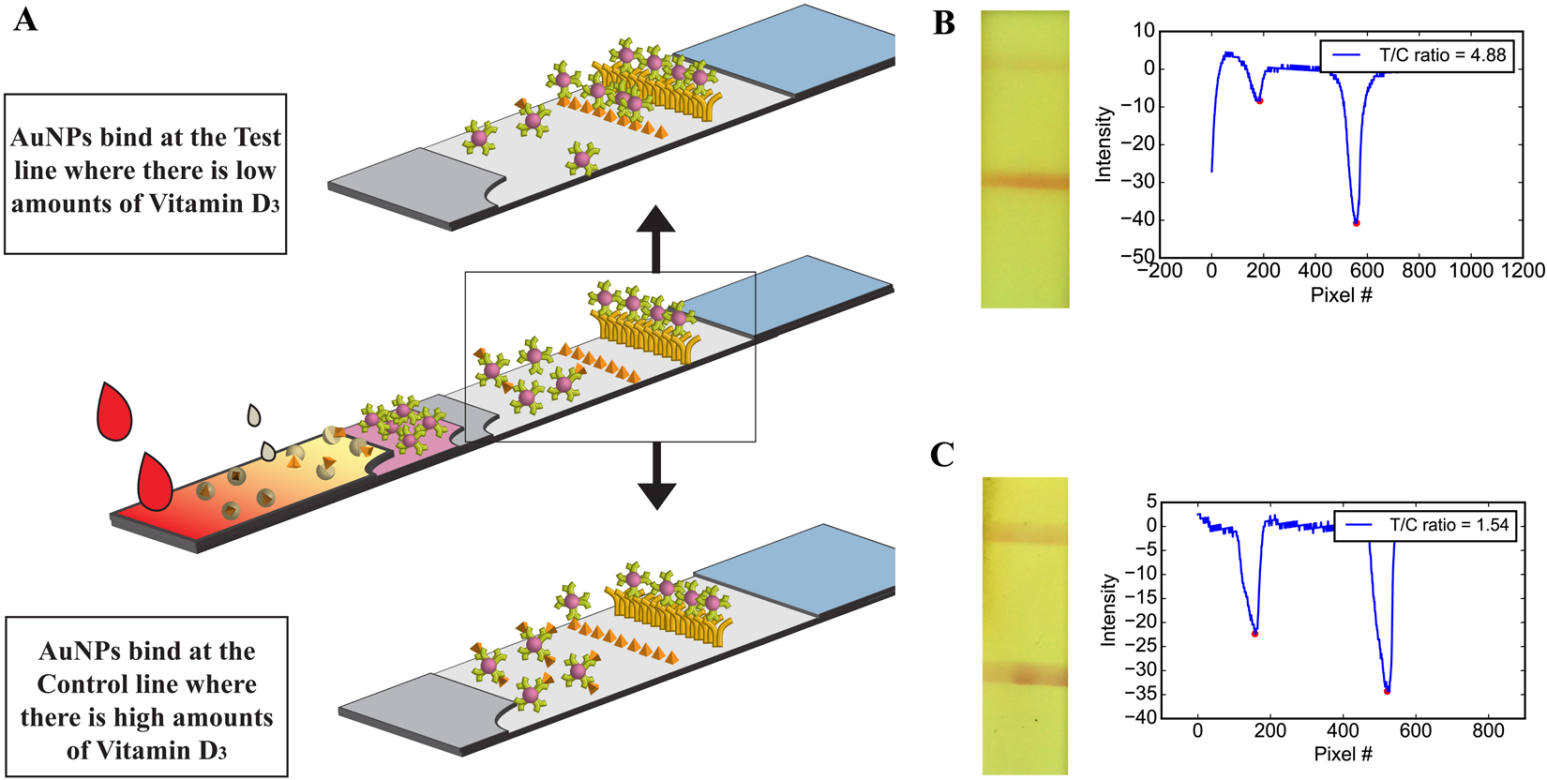
Vitamin D_3_ Lateral Flow Assay. (**A**) Image and schematic of the 25(OH)D_3_ strip architecture and components. (**B**) Image and intensity plot of a participant with low Vitamin D_3_ and high T/C ratio (**C**) Image and intensity plot of a participant with healthy levels of 25(OH)D_3_ and low T/C ratio.

Vitamin D_3_ diagnostic test protocol: The system consists of a vitamin D_3_ diagnostic test, custom made cassette that houses the test and a portable reader (TIDBIT) (See Fig. 1). The TIDBIT has been designed so that when the cassette is inserted into the device, it is aligned with a CMOS camera that automatically captures an image of the test area. The TIDBIT can be operated via a smartphone or computer application and can be controlled with the help of the Nutriphone application. Once the image is taken, a custom python script automatically crops the image and deduces the ratio of intensities of the test line to the control line, or T/C ratio (See Fig. 2B and C). The T/C ratio is correlated to a value of 25(OH)D_3_ by using a calibration curve, that is determined prior to testing by analyzing samples of known 25(OH)D_3_ values.

To run a vitamin D_3_ test, the user starts the Nutriphone application on the device of choice and follows the instructions. First the user applies a small amount (4 µL) of elution buffer on the edge of the sample pad. The user then collects 40 µL of blood from a finger prick and places it onto the sample collection pad. After allowing for the blood to separate into serum and mix with the elution buffer, the user is prompted by the smartphone application to apply 15 µL of running buffer for the filtered serum to enter the conjugate pad. After another short period of incubation, the user is again prompted to add 25 µL of running buffer to start the test. Following this step, the user must place the cassette in the TIDBIT, which automatically images and analyzes the test strip when the test and control lines are fully developed. Briefly, a grayscale image is taken using the TIDBIT and processed by a python script to filter out the noise using a Gaussian filter. The noisy 2D image is filtered and converted to the grayscale, followed by a median filtering process to convert the 2D image into a 1D array, reducing the task to analyzing a 1D digital signal. The test and control lines of concentrated AuNP – anti 25(OH)D_3_ are detected as local minima on the intensity plot. The respective peak values can be used to calculate a T/C ratio.

### Design and implementation of a protein elution buffer

Vitamin D binding protein interferes with the capture and quantification of 25(OH)D_3_ via traditional immunoassay techniques. Commercial tests such as the DiaSorin RIA call for mixing the sample with 10X the amount of acetonitrile, an organic solvent, in order to liberate all 25(OH)D_3_ in the sample from the binding protein^[Bibr CR23][Bibr CR23]^. This method cannot be directly translated for application with a low-cost point-of-need lateral flow platform for several reasons. Firstly, organic solvents cause lysis of red blood cells^[Bibr CR24][Bibr CR24]^, making it unsuitable for use with blood samples; the sample must be first spun down into serum via centrifugation, a process that is difficult to perform in point-of-need settings. Secondly, if not neutralized or removed prior to running the test, acetonitrile could negatively affect the conjugated gold nanoparticles and the dispensed reagents on the test and control lines as the antibodies present are prone to denaturation themselves. Finally, adding large amounts of organic solvent is particularly unsuitable for lateral flow platforms, as they may adsorb to the nitrocellulose membrane and alter its wetting properties^[Bibr CR25][Bibr CR26]^. It was therefore necessary to develop an elution buffer that could denature binding protein in the sample without lysing blood cells while maintaining compatibility with the nitrocellulose membrane, the conjugated nanoparticles and the dispensed reagents.

**Figure 3:**
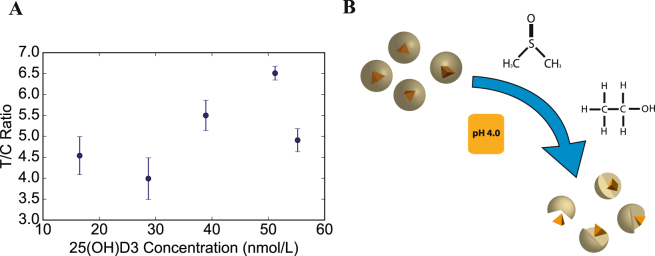
Elution Buffer. (**A**) Elution of 25(OH)D_3_ from binding protein with the help of organic solvents and low pH buffer and (**B**) Calibration curve obtained from analyzing samples without the use of elution buffer. As seen, there is no relationship between [25(OH)D_3_] and T/C ratio without the use of the elution buffer.

In order to perform the separation of 25(OH)D_3_ from its binding protein, our diagnostic test takes advantage of carefully optimized organic solvents and low pH buffers, which are known to be effective at denaturing proteins. After assessing a number of possible combinations, we decided on a combination of Dimethyl sulfoxide (DMSO), ethanol and a low pH acetate buffer. We hypothesize that DMSO and ethanol are able to unfold vitamin D binding protein while also serving as organic solvents to stabilize the liberated 25(OH)D_3_^[Bibr CR26][Bibr CR25]^. This unfolding effect is further enhanced in the presence of a low pH buffer as most proteins undergo destabilization at low pH^[Bibr CR25][Bibr CR26]^. As seen in Fig. 3B, there is no relationship between [25(OH)D_3_] and the T/C ratio when samples are tested for vitamin D_3_ without the use of any elution buffer.

### Vitamin D_3_ diagnostic test architecture and principles

The vitamin D_3_ diagnostic test seen in Fig. 2 consists of a: blood filtration membrane, a conjugate pad that dry stores gold nanoparticle conjugates (AuNP – anti 25(OH)D_3_), a spacer pad that allows for incubation of the sample with the elution buffer and the conjugates, a nitrocellulose membrane that houses immobilized 25(OH)D_3_-BSA and secondary antibodies and a cellulose fiber absorbent pad respectively. When applicable, the blood filtration membrane can be substituted for a cellulose fiber pad to perform testing in human serum. Our test strip adopts a competitive type architecture as 25(OH)D_3_ is unable to bind to more than one antibody at a time due to its small size (~350 Daltons).

The architecture of the diagnostic test was designed in order to prevent any need for specialized sample preparation. Prior to starting the test, the user dispenses a small amount of elution buffer onto the front edge of the blood filtration membrane to prevent the interaction of the elution buffer and the red blood cells present in the blood sample. Once the user dispenses the blood on the sample pad, it automatically flows forward through the filtration paper and separates into serum while simultaneously mixing with the elution buffer. This causes bound 25(OH)D_3_ to be released from the binding protein present in the sample. After the first instance of running buffer is applied, the sample enters the conjugated pad and the unbound 25(OH)D_3_ interacts with the AuNP-anti 25(OH)D_3_ conjugates. This step is critical for the proper functioning of the assay. Based on the amount of 25(OH)D_3_ in the sample, there is a competitive interaction between the immobilized 25(OH)D_3_-BSA on the nitrocellulose and the 25(OH)D_3_ in the sample for binding sites on the AuNP-antibody conjugates as the sample flows through the nitrocellulose. As seen in Fig. 2B, an individual with vitamin D_3_ deficiency will observe a T/C ratio that is high as most binding sites on the conjugates will be empty and free to bind to the immobilized 25(OH)D_3_. Conversely, an individual with healthy levels of 25(OH)D_3_ will observe a T/C ratio that is low as most of the binding sites on the conjugates are occupied with 25(OH)D_3_ from the sample which prevents them from binding at the test line (Fig. 2C).

### 25(OH)D_3_ quantification in standard solution and commercial calibrators

**Figure 4:**
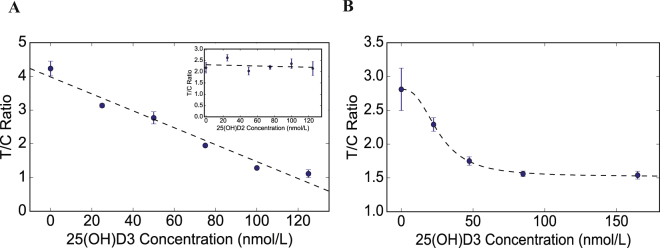
Calibration curve in standards. (**A**) T/C ratios of the colorimetric signals at different 25(OH)D_3_ concentration in standard buffers. T/C ratio = a[25(OH)D_3_] + b, where a = −0.0251 and b = 3.983. Linear logistic curve was fitted with R^2^ = 0.95. The inset demonstrates a calibration curve performed with standard buffers that were spiked with 25(OH)D_2_ to verify cross-reactivity. A linear logistic curve was fitted with R^2^ = 0.0059 indicating no dependence of T/C ratio on [25(OH)D_2_] (**B**) T/C ratios of the colorimetrics signals at different 25(OH)D_3_ concentrations in commercial serum based calibrators. T/C ratio $={\boldsymbol{d}}+\frac{{\boldsymbol{a}}-{\boldsymbol{d}}}{1+{(\frac{[25({\boldsymbol{OH}}){\boldsymbol{D}}3]}{{\boldsymbol{c}}})}^{{\boldsymbol{b}}}}$, where a = 2.81, b = 2.63, c = 26.2 and d = 1.52. A four parameter logistic curve was fitted with R^2^ = 0.99. At each concentration two strips were used and the maximum and minimum deviation from the average is shown as error bars.

In Fig. 4A, we demonstrate that the T/C ratio can be correlated to a 25(OH)D_3_ concentration in standard solutions. At each concentration, two strips were used and the maximum lower and upper deviations from the average T/C values were shown. The coefficient of variation of our assay ranges from 1.7% at 100 nmol/L to 14.7% at 125 nmol/L. We then fitted a linear curve such that [25(OH)D_3_] = f(T/C). We also tested the performance our assay with standard solutions that were spiked with 25(OH)D_2_ in order to evaluate the cross-reactivity of the assay (inset in Fig. 4A). To verify the accuracy of the assay in serum, 25(OH)D_3_ levels were also quantified in commercially available serum based standards (ChromSystems Inc). The calibrators are based on pooled human serum and contain artificially spiked values of 25(OH)D_3_ that lie in the physiologically relevant range. As seen in Fig. 4B, our assay is able to reliably distinguish between a deficient, insufficient and healthy sample. These standards did not require the use of the elution buffer as these samples were prepared by artificially introducing 25(OH)D_3_ into the sample. The coefficient of variation of our assay ranges from 2.63% at 85 nmol/L to 11.2% at 0 nmol/L.

### Vitamin D_3_ deficiency testing in human serum

**Figure 5:**
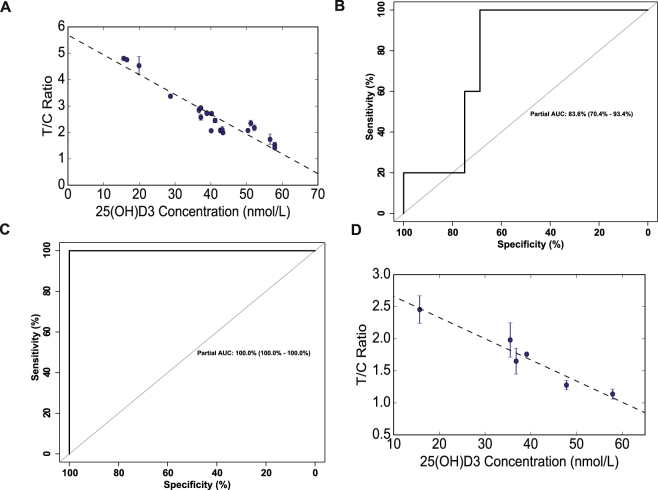
Analysis of human trial samples (**A**) Linear calibration curve for 21 human serum samples with R^2^ = 0.91. T/C ratio = a[25(OH)D_3_] + b where a = −0.075 and b = 5.689 (**B**) ROC curve obtained with Delong method with n = 300000 and auc 0.836 at a cutoff of 50nmol/L (**C**) ROC curve obtained from Delong method with n = 300000 and auc 1 at a cutoff of 30 nmol/L (**D**) Calibration curve from 6 human finger stick blood samples with R^2^ = 0.94. T/C ratio = a[25(OH)D3] + b where a = −0.033 and b = 2.9925.

Our assay was used to quantify the vitamin D_3_ levels in human serum samples that were collected from a human trial to demonstrate the assay’s applicability in the clinical setting (Fig. 5A,B and C). Native serum samples required the use of elution buffer to separate 25(OH)D_3_ from vitamin D binding protein. As expected of our competitive type architecture, the T/C ratio for a participant with vitamin D deficiency appears to be significantly greater than that of a participant with vitamin D insufficiency. Actual serum 25(OH)D_3_ values were quantified using tandem liquid chromatography/mass spectrometry (LC/MS-MS). Figure 5B shows a ROC curve for the serum tests based on a linear calibration curve that was determined from the same batch of test strips (Fig. 5A). We used the DeLong method^[Bibr CR27][Bibr CR27]^ to obtain an asymptotically exact method to evaluate the area under curve (auc) using 300,000 iterations with a cutoff of 50 nmol/L. A similar method was used to evaluate the performance of the assay at a cutoff of 30 nmol/L^[Bibr CR28][Bibr CR28]^ that can be seen in Fig. 5C. Diagnostic accuracy, defined as the proportion of correctly classified subjects to all subjects, was evaluated as 90.5% and 100% for the two cutoffs respectively. Both cutoffs are based on recommendations made by the Institute of Medicine. The root mean square error was assessed to be 5.4 nmol/L. The coefficient of variation ranged from 0.05% at 43 nmol/L to 16.6% at 57 nmol/L.

### Vitamin D_3_ deficiency testing in finger prick blood samples

We used our assay to quantify vitamin D_3_ levels in finger prick blood from volunteers after obtaining informed consent, as per a protocol approved by the Institutional Review Board at Cornell University. Samples were run and analyzed at the site of the trial. Figure 5D demonstrates a calibration curve for the sample set with R^2^ value of 0.94. From Fig. 5D, it is apparent that our assay demonstrates a linear correlation between the T/C ratio and [25(OH)D_3_]. Additionally, by observing the change in T/C ratio, we are able to demonstrate that our test can distinguish between a severely deficient, deficient and insufficient participant with reasonable certainty. The coefficient of variation ranged from 0.5% at 39 nmol/L to 17.2% at 48 nmol/L.

## Discussion

In this work, we demonstrate a rapid and easy to use microfluidic platform for the assessment of vitamin D status. We showed the quantification of 25(OH)D_3_ in the physiologically relevant range within minutes. This was achieved by using a competitive type lateral flow assay that incorporates a novel elution buffer which separates 25(OH)D_3_ from its binding protein on the strip, therefore eliminating the need for tedious sample preparation. The assay produces a colorimetric image that can be analyzed to obtain a T/C ratio that can be correlated to a concentration of 25(OH)D_3_ in the sample (serum or blood). We further validate the system by quantifying levels in trivial amounts of human serum and a finger prick of blood. Given the limitations of traditional clinical diagnostic methods, our assay has the potential to make an immediate impact in the point-of-need setting as an accurate tool for the assessment of vitamin D_3_ status.

Our work presented here is the first quantitative, point-of-need diagnostic test for the assessment of vitamin D_3_ status and represents a critical step towards transitioning vitamin D deficiency testing out of the lab and into the hands of individuals. Unlike commercial platforms that have been developed for micronutrient testing, our test is able to process finger stick blood, denature vitamin D binding protein *in situ* and quantify levels of 25(OH)D_3_ in the sample in a few minutes without the need for expensive equipment. These unique traits coupled with a high diagnostic accuracy (90.5%) make our test sufficient for application at the point of need. Our assay is designed specifically for assessing 25(OH)D_3_ status in the deficient range ( < 50 nmol/L) as this is where differentiation is most important. However, we have also demonstrated that our test is able to reliably quantify concentrations in the complete physiologically relevant range (0–150nmol/L). Since our assay is unable to detect 25(OH)D_2_, the quantitative ability is limited when used in settings where ergocalciferol is the preferred supplement. However, the recent uptake in vitamin D_3_ supplementation suggests that our diagnostic test is practical for use in many point-of-need settings^29,30^. Given the minimal amount of expertise needed to run the assay, our test enables individuals to evaluate their own vitamin D status irrespective of their medical training or knowledge. The low cost and portability of the testing platform will also make monitoring and evaluation of the efficacy of nutritional interventions feasible in both research and programmatic settings. Furthermore, we have developed a protein elution buffer that could be adopted to improve several other diagnostic tests that require specialized sample preparation because of the presence of a binding protein (vitamins A, B_7_ and B_12_).

## Materials and Methods

The monoclonal anti-25(OH)D_3_ IgG produced in mouse (GenScript Inc) was conjugated with 40 nm Gold Nanoparticles using the InnovaCoat Gold Conjugation Kit (Innova Biosciences Ltd). For the conjugation, 0.23 µg AuNP in freeze dried form was mixed with 1ug anti 25(OH)D_3_ IgG in 0.01 M amine free phosphate buffer saline at pH 7.4. The anti-25(OH)D_3_ IgG is attached to the surface of AuNP via lysine residues during a 15-minute incubation and the reaction was terminated by adding 0.1 M tris-buffered saline (TBS) with 0.1% Tween 20. To remove excess antibodies, DI water was added in 10 times the volume of the conjugate mixture as centrifuged at 9000 g for 10 min. Upon removal of the supernatant, the final gold nanoparticle concentration was reconstituted in 0.01 M TBS containing 2% BSA and the final optical density was verified by using Spectramax 384 to measure absorbance of the conjugates at a wavelength of 530 nm. The conjugates were stored at 4 °C until use. Conjugate pads were prepared by first diluting the conjugates to roughly 0.7–0.8 OD (depending on application) in a conjugate buffer (2 mM Borate buffer with 5% sucrose) which contained appropriate preservatives. Glass Fiber conjugate pads (EMD Millipore) with 30cmx5 cm dimensions were soaked in diluted conjugate solution for 2 minutes followed by drying at 37 °C for a minimum of 12 h.

### Vitamin D_3_ lateral flow assay preparation

Hi Flow Plus 180 membrane Cards (EMD Milipore) were used as the assay platform. The membrane cards have a 2mm clear polyester film backing that contains the nitrocellulose membrane and the adhesive that allows for assembly of conjugate and sample pads. The nitrocellulose part of the assay extends for 2.5 cm in length and has a nominal capillary flowrate of 45 seconds/cm. This is the slowest flow rate offered by the manufacturers and was chosen to allow for maximum reaction time between the sample and the conjugates due to the relatively low detection range. The test and control lines were prepared by using a Lateral Flow Reagent Dispenser (Claremont Bio) to dispense 0.4 mg/mL of anti-mouse IgG produced in goat and 1 mg/mL of 25(OH)D_3_-BSA. The two lines are separated by 3mm and have line widths of 1mm. These lines were dispensed using the Legato 200 Dual Syringe Pump (Claremont Bio) at 6.7 µL/min. Once the reagents were dispensed, they were dried for a minimum of 12 hours at 37 °C. The final assay was assembled by first applying the spacer pad onto the bottom of the nitrocellulose portion with 2–3mm overlap followed by a dried conjugate pad. The sample pad, which is the final component, varies based on the sample that is to be tested. Cellulose pads (EMD Milipore) were used for serum samples and FR1 blood filtration membranes (MDI membrane technologies) were used for blood samples. In both cases, the sample pad was attached below the conjugate pad with 2mm overlap. The FR-1 membrane has a thickness of 0.35 mm and capacity of 30 µL/cm^2^ and measured to be 30 cm × 2.5 cm while the Cellulose pad measured to be 30 cm × 1 cm. Another cellulose fiber sample pad was attached at the top of the nitrocellulose card to serve as an absorbent pad to collect any waste from the assay. The assembled assay was then cut into 4mm strips using a rotary paper trimmer. The strips were then kept sealed in a plastic container and wrapped in aluminum foil in a humidity controlled chamber (Secador) for a few days to prevent adverse effects of exposure to varying temperature and humidity. Test strips remain integral for up to several weeks while stored in these conditions. However, their shelf life can be extended up to a year if they are sealed in light safe mylar pouches and stored at room temperature.

### Elution buffer preparation

A combination of organic solvents and low pH buffers was used to ensure the separation of vitamin D binding protein and 25(OH)D_3_ on strip. We mixed 4.7 parts of 1:1 mixture of Dimethyl sulfoxide (VWR) and Pure Ethanol (Acros Organics) with 1 part of pH 4.0 acetate buffer (VWR). In the case of finger prick blood testing, 4 µL of this mixed buffer was added to the area of overlap between the blood filtration membrane and the conjugate pad to allow for the filtered serum to interact with the elution buffer prior to the reaction with the conjugates. Elution buffer cannot be added directly to whole blood as the organic solvents cause lysing of blood cells. As for serum, the same amount of elution buffer was mixed in with the sample in a plastic 1.5 mL micro-centrifuge vial prior to applying the sample to the strip.

### Vitamin D_3_ test strip protocol and human trials

Capillary tubes were used for the collection of constant amount of blood from the participants. First, 4 µL of elution buffer was pipetted onto the area of overlap between the sample pad and the conjugate pad. This allows for the filtered serum to interact with the elution buffer prior to the conjugates. Elution buffer is added prior to the interaction of the sample with the AuNP-anti 25(OH)D_3_ conjugates to avoid affecting the performance of the gold nanoparticles. Nanoparticle aggregation was observed when the conjugates are mixed directly with elution buffer. Additionally, adding elution buffer to the sample prior to incubation reduces the adverse effects of the elution buffer on the conjugates. After allowing for 4 minutes for the sample to filter into serum, 15 µL of running buffer is added to flow the sample and elution buffer mixture into the conjugate pad. After allowing for an additional four minutes of incubation, 25 µL of running buffer is added to start the flow onto the nitrocellulose membrane. At this point, the user inserts the cartridge into the imaging device. The imaging device automatically images the diagnostic test when the test and control lines are fully developed.

The human trials were approved by the Institutional Review Board for Human Subjects at Cornell University and carried out in accordance with their regulations. Informed consent was obtained from all participants prior to testing. For the trial, the participants were subjected to a finger prick (UniStik) for a drop of blood which was collected using a capillary tube and dispensed onto the sample pad. A trained and certified phlebotomist then drew 5 mL of blood via venipuncture which was used for tandem mass spectrometry analysis. Following 1 hour incubation at room temperature, serum was separated by centrifugation at 2000 g for 10 min. Serum 25(OH)D levels were characterized using tandem mass spectrometry (LC/MS-MS).

### TIDBIT and image processing

Test strips were imaged using a portable imaging device designed specifically for colorimetric detection and quantification. The device consists of a 5 megapixel 1080p HD CMOS camera (Rasbperry Pi), focusing lens and LEDs (Thorlabs Inc), raspberry Pi computer board, rechargeable lithium ion battery pack (Adafruit Industries) and slide out cassette tray, all surrounded by a 3D printed light tight container. Once the cassette is inserted into the imaging device, it is automatically imaged and processed using a custom python script.

### Data availability

All data generated or analyzed during the current study are available from the corresponding author on reasonable request.
